# AVE0991, a Nonpeptide Compound, Attenuates Angiotensin II-Induced Vascular Smooth Muscle Cell Proliferation via Induction of Heme Oxygenase-1 and Downregulation of p-38 MAPK Phosphorylation

**DOI:** 10.1155/2012/958298

**Published:** 2012-02-26

**Authors:** Chen Sheng-Long, Wu Yan-Xin, Huang Yi-Yi, Fang Ming, He Jian-Gui, Chen Yi-Li, Xia Wen-Jing, Ma Hong

**Affiliations:** ^1^Acute and Intensive Care Department, Guangdong General Hospital, Guangdong Academy of Medical Science, Guangzhou 510080, China; ^2^Department of Gynaecology, First Affiliated Hospital of Sun Yat-sen University, Guangzhou 510080, China; ^3^Department of Cardiology, First Affiliated Hospital of Sun Yat-sen University, Guangzhou 510080, China

## Abstract

The nonpeptide AVE0991 is an agonist of the angiotensin-(1–7) (Ang-(1–7)) Mas receptor and is expected to be a putative new drug for treatment of cardiovascular disease. However, the mechanisms involved in the antiproliferative effects of AVE0991 are not fully understood. We saw that the compound attenuated proliferation in an angiotensin II-induced rat vascular smooth muscle cells (VSMC) proliferation model. Moreover, treatment with AVE0991 (10^−5^ mol/L or 10^−7^ mol/L) significantly attenuated reactive oxygen species (ROS) production, phosphorylation of p38 MAPK, and dose-dependently (10^−8^ to 10^−5^ mol/L) inhibited Ang II-induced VSMC proliferation. Meanwhile, heme oxygenase-1 (HO-1) expression increased in the AVE0991 + Ang II group (10^−5^ mol/L or 10^−6^ mol/L). However, the beneficial effects of AVE0991 were completely abolished when the VSMC were pretreated with A-779 (10^−6^ mol/L). Furthermore, treatment with the HO-1 inhibitor ZnPPIX attenuated the inhibitory effect of AVE0991 on Ang II-induced p38MAPK phosphorylation. These results suggest that AVE0991 attenuates Ang II-induced VSMC proliferation in a dose-dependent fashion and that this effect is associated with the Mas/HO-1/p38 MAPK signaling pathway.

## 1. Introduction

Angiotensin-(1–7) (Ang-(1–7)) is a potent, endogenous effector hormone of the renin angiotensin system (RAS) pathway. It can be formed directly from Ang I or Ang II, or indirectly from Ang I, where Ang-(1–9) is produced in an intermediate step [1]. Santos et al. demonstrated that a G-protein-coupled receptor is the specific Ang-(1–7) receptor in *Mas*-deficient mice [2]. Previous studies have shown that Ang-(1–7) has opposite effects to Ang II, which induces myocardial hypertrophy and ultimately cellular proliferation [3, 4]. A-779 is a selective antagonist to the Ang-(1–7) Mas receptor and can therefore prevent effects of Ang-(1–7) by preventing ligand/receptor interactions [5].

Because the Ang-(1–7) peptide is not resistant to proteolytic enzymes, its clinical application is limited. Wiemer et al. have shown that a nonpeptide compound (AVE0991) produces similar effects as Ang-(1–7) in biological systems [6]. Previous studies have shown that AVE0991 ameliorated hepatic fibrosis in the bile-duct-ligated rat model and improved myocardial fibrosis induced by isoproterenol in male Wistar rats [7, 8]. AVE0991 is an orally available compound that has a wider clinical application and thus could be a putative new drug for the treatment of cardiovascular disease. However, the antiproliferative mechanisms of AVE0991 are still not fully understood.

Numerous studies have shown that Ang II plays a critical role during proliferation in vascular smooth muscle cells (VSMCs) [9–12]. The molecular and cellular mechanisms underlying the Ang II-dependent processes in vascular remodeling have not been fully elucidated. However, the mitogen-activated protein kinase (MAPK) cascade, particularly the p38 MAP kinase, may play a role in mediating responses that are related to cell growth and differentiation [13]. Ang II is a well-known activator of this signaling pathway.

Heme oxygenases (HOs), which catalyze the breakdown of heme to equimolar quantities of carbon monoxide (CO), biliverdin, and ferrous iron, are the rate-limiting enzymes in heme degradation. These enzymes have antioxidative and anti-inflammatory effects, and several studies have suggested that HO-1 has a cytoprotective role [14, 15]. Additionally, many studies have indicated that HO-1 has a beneficial antiproliferative effect in VSMCs and that this effect can be abolished by the HO-1 inhibitor ZnPPIX [16].

Moreover, Ang II-stimulated growth of VSMCs has an essential redox-sensitive component that is mediated by activation of MAPK-dependent signaling pathways, while HO-1 attenuates the Ang II-induced damage in VSMCs [17]. In addition, Sun et al. have shown that HO-1 attenuated Ang II-induced VSMCs proliferation via inhibiting the expression of MAPK [18]. Hence, we hypothesize that the HO-1/p38 MAPK signaling pathway may be involved in the inhibition of VSMCs proliferation.

The objectives of this study were to determine whether the nonpeptide compound AVE0991 could inhibit Ang II-induced VSMCs proliferation and if the HO-1/p38 MAPK signal pathways are involved in the AVE0991-mediated effects.

## 2. Materials and Methods

The investigation was carried out according to the Guide for Care and Use of Laboratory Animals that was published by the US National Institutes of Health (NIH Publication no. 85-23, revised in 1996).

### 2.1. Materials

The peptides Ang II and the Ang-(1–7) Mas receptor antagonist A779 were obtained from Bachem (King of Prussia, PA, USA). The *β*-actin monoclonal antibody and HO-1 inhibitor ZnPPIX were obtained from Sigma-Aldrich (St. Louis, MO, USA). Dulbecco's modified Eagle medium (DMEM) and trypsin were obtained from Invitrogen (Carlsbad, CA, USA). [^3^H]Thymidine (20 Ci/mmol) was obtained from the Atomic Energy Institute of China (Wuhan, China). Fetal bovine serum (FBS) was purchased from the Sijiqing Company (Hangzhou, China). AVE0991 was kindly provided by Dr. Juergen Puenter of Aventis Pharma (Frankfurt, Germany) and male Sprague-Dawley rats (10 to 12 weeks old) were obtained from the Experimental Animal Facility of Sun Yat-sen University, China.

### 2.2. Cell Culture and Experimental Designs

**Figure 1:**
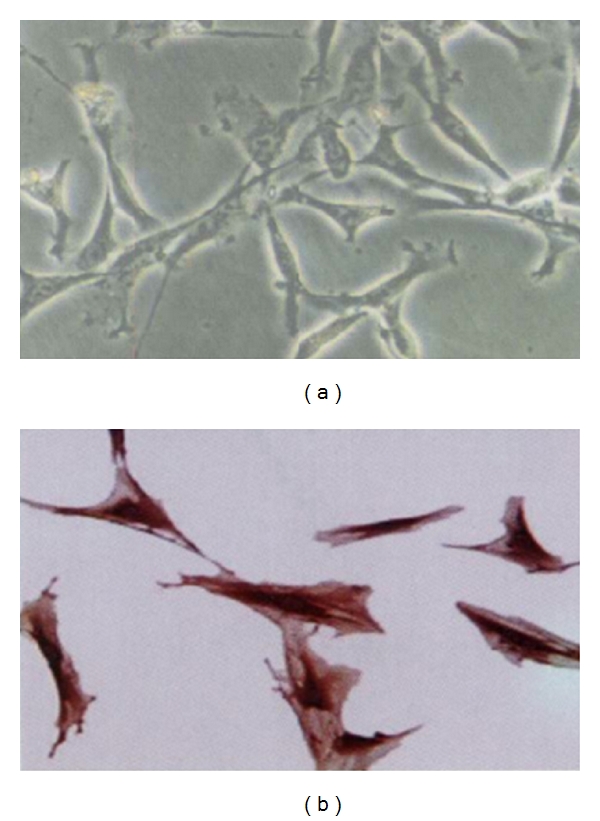
The analysis of cultured VSMCs in morphology and identification. (a) The morphology of cultured VSMCs detected by phase-contrast microscope. Magnification of light microscopy images is ×100. (b) SM-*α* actin immunocytochemical staining of cultured VSMCs. Magnification of light microscopy images is ×400.

VSMCs were isolated from the thoracic aorta of 10- to 12-week-old male Sprague-Dawley rats by an explant culture method. The cells were seeded in DMEM/Ham's F-12 (1 : 1) that was supplemented with 10% FBS, 100 *μ*g/mL penicillin, and 100 U/mL streptomycin (Invitrogen). The cell preparations were cultured at 37°C in a humidified atmosphere with 5% CO_2_. The culture purity was assessed by immunostaining with a monoclonal antibody against smooth muscle *α*-actin, followed by an anti-mouse fluorescein-conjugated goat IgG antibody. Using these methods, the purity of VSMCs reached more than 98%. Cells between passages 4 and 7 were used for all of the experiments [19] (Figure 1).

The cells were rendered quiescent by serum starvation for 36 hours before ROS, HO-1, and p38 phosphorylation detection, and for 48 hours before thymidine incorporation experimentation. The VSMCs were treated according to the following experimental protocols.

**Protocol 1**

Effect of various concentrations of AVE0991 on the VSMCs [^3^H]thymidine incorporation efficiency.

The cells were pretreated with Ang II for 24 hours and then followed by AVE0991 (10^−5^ mol/L, 10^−6^ mol/L, 10^−7^ mol/L, or 10^−8^ mol/L) for 24 hours.

**Protocol 2**

Effects of AVE0991 on Ang II-induced VSMC proliferation, ROS production, HO-1 protein, and p38 phosphorylation expression.

For Ang II group, the cells were treated with Ang II for 48 hours. For Ang II + AVE0991-H (10^−5^ mol/L) group and Ang II + AVE0991-L (10^−7^ mol/L) group, the cells were pretreated with Ang II for 24 hours and followed by AVE0991 for 24 hours. And for Ang II + AVE0991-H (10^−5^ mol/L) + A-779 group, the cells were pretreated with A-779 for 30 min, followed by Ang II for 24 hours, and then AVE0991 for 24 hours.

**Protocol 3**

Effects of AVE0991 on Ang II-induced VSMCs proliferation, ROS production, and p38 phosphorylation expression with pretreatment of HO-1 inhibitor ZnPPIX.

For Ang II group, the cells were treated with Ang II for 48 hours. For Ang II + AVE0991 (10^−5 ^mol/L) group, the cells were pretreated with Ang II for 32 hours and followed by treatment with AVE0991 for 16 hours. For Ang II + AVE0991 (10^−5^ mol/L) + A-779 group, the cells were pretreated with A-779 for 30 min, then treated with Ang II for 32 hours, and then followed by treatment with AVE0991 for 16 hours. And for Ang II + AVE0991 (10^−5^ mol/L) + ZnPPIX group, the cells were treated in the following order: Ang II for 24 hours, HO-1 inhibitor ZnPPIX for 8 hours, and AVE0991 for 16 hours.

### 2.3. [^3^H]Thymidine Incorporation

De novo DNA synthesis was measured via incorporation of tritiated thymidine by VSMCs that were grown in 24-well culture plates. The cells were plated at a density of 2000 cells/well and subconfluent monolayers were made quiescent by serum starvation for 48 h. The monolayers were then treated with or without Ang II for 48 h and were subsequently treated with various concentrations of AVE0991, A-779, and/or ZnPPIX. During the last 24 h, 0.25 *μ*Ci of [^3^H]thymidine/mL culture medium was added to the growth medium. The incorporation of [^3^H]thymidine was determined after precipitation of acid-insoluble material with ice-cold 5% trichloroacetic acid. The acid-insoluble material was dissolved in 0.25% NaOH and counted in a liquid scintillation spectrometer in the presence of 5 mL Ecolite.

### 2.4. Detection of ROS Production

VSMCs were preloaded with 30 *μ*mol/L 2′-7′-dichlorofluorescein diacetate at 37°C for 75 min and were treated with the indicated agents for an additional 2 h in serum-free medium. After washing with Hanks, the cells were lysed with Tris-HCl (10 mmol/L, pH 7.4, containing 0.5% Tween-20) and were centrifuged at 10,000 g for 10 min. The fluorescence intensity of the supernatants was determined with a spectrofluorometer [20].

### 2.5. Western Blotting

The cytosolic proteins were extracted, and the total protein concentration was determined with a BCA Protein Assay kit (Pierce, Rockford, IL, USA). Thirty micrograms of crude protein extract was loaded on to a 10% SDS-polyacrylamide gel and transferred to a polyvinylidene fluoride membrane. The membrane was blocked for 2 h at room temperature and incubated with an anti-HO-1 (1 : 1000 dilution; Santa Cruz Biotechnology, Santa Cruz, CA, USA), anti-phosphorylated p38 or p38 (1 : 800 dilution; Cell Signal, Beverly, MA, USA), or anti-GAPDH (1 : 10 000 dilution; Boshide, Wuhan, China) antibody diluted in Tris-buffered saline/Tween-20 (TBS-T). After washing, the membranes were subsequently incubated for 1 h with rabbit anti-mouse or goat anti-rabbit secondary antibodies diluted 1 : 2000 or 1 : 3000, respectively, in TBS-T. The bands were visualized with an ECL kit (GE Healthcare, Chalfont St Giles, Bucks, UK) according to the manufacturer's instructions, and GAPDH was used as a loading control. The results were analyzed with a gel image analysis system (Bio-Rad, Richmond, CA, USA).

### 2.6. Statistical Analysis

The data are expressed as the mean ± standard deviation (SD). Differences between groups were evaluated by two-tailed unpaired Student's *t*-test, and a value of *P* < 0.05 was interpreted as being statistically significant. Statistical analyses were performed using SPSS 13.0 statistics software (SPSS Inc., Chicago, IL, USA).

## 3. Results

### 3.1. Effects of AVE0991 on Ang II-Induced VSMC Proliferation and ROS Production

**Figure 2:**
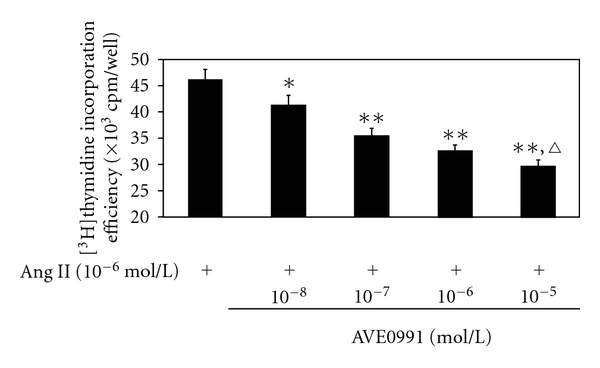
The effect of various concentrations of AVE0991 on the VSMCs [^3^H]thymidine incorporation efficiency when stimulated with Ang II. *P < 0.05; **P < 0.01 versus control group; ^*▵*^ *P* < 0.05  versus Ang II + AVE0991 (10^−6 ^mol/L) group; *n* = 8.

Firstly, we evaluated the inhibition effect of AVE0991 on Ang II-induced VSMCs proliferation. Ang II (10^−6^ mol/L) and AVE0991 (10^−5^ mol/L, 10^−6^ mol/L, 10^−7^ mol/L, or 10^−8^ mol/L) were added to culture fluid. The [^3^H]thymidine incorporation efficiency of VSMCs was inhibited by AVE0991 in a dose-dependent manner (Figure 2).

**Figure 3:**
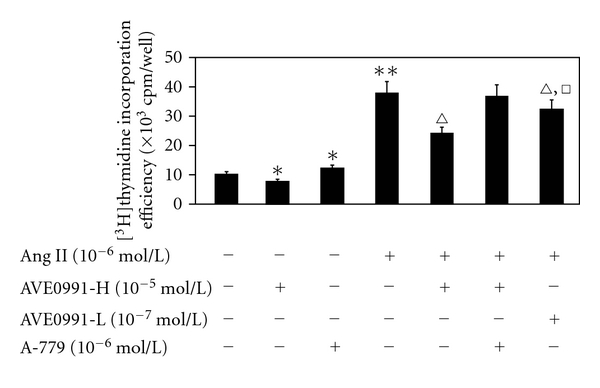
The effect of AVE0991 on the [^3^H]thymidine incorporation efficiency of VSMCs that were stimulated by Ang II. *P < 0.05; **P < 0.01 versus control group; ^*▵▵*^ *P* < 0.01 versus Ang II group; ^□^ *P* < 0.05 versus Ang II + AVE0991-H group; *n* = 8.

Secondly, treatment with 10^−6^ mol/L of Ang II significantly increased the [^3^H]thymidine incorporation efficiency of VSMCs compared to the control group (*n* = 8, *P* < 0.01) (Figure 3). When the VSMCs were treated with the same dose of Ang II combined with AVE0991 (10^−5^ mol/L and 10^−7^ mol/L), the [^3^H]thymidine incorporation efficiency decreased significantly when compared to the Ang II group (*n* = 8, *P* < 0.01), although the efficiency was still higher than the control group. Nevertheless, when the cells were treated with Ang II + AVE0991 + A-779 (10^−6 ^mol/L), this AVE0991-mediated effect which inhibited [^3^H]thymidine incorporation efficiency of VSMCs was abolished. However, when the VSMCs were treated with 10^−5^ mol/L AVE0991 or 10^−6^ mol/L A-779, both reagents affected the [^3^H]thymidine incorporation efficiency of the VSMCs. Compared to the control group, the [^3^H]thymidine incorporation efficiency was decreased when the cells were treated with AVE0991 alone (*n* = 8, *P* < 0.05). Conversely, the efficiency increased when the cells were treated with A-779 alone (*n* = 8, *P* < 0.05).

**Figure 4:**
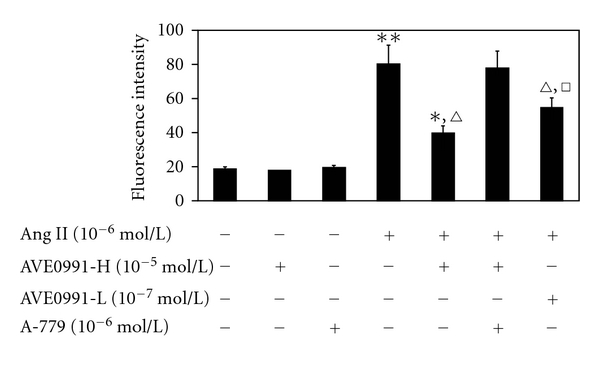
The effect of AVE0991 on Ang II-stimulated ROS production in VSMCs. *P < 0.05; **P < 0.01 versus control group; ^*▵*^ *P* < 0.01 versus Ang II group; ^□^ *P* < 0.05 versus Ang II + AVE0991-H group; *n* = 8.

We sought to determine whether AVE0991 inhibition of Ang II-stimulation has any effect on ROS production afterwards. The ROS level was significantly increased in the Ang II group (10^−6^ mol/L) compared to the control group (Figure 4). AVE0991 significantly inhibited Ang II-stimulated ROS expression, especially in the Ang II (10^−6^ mol/L) + AVE0991-H (10^−5^ mol/L) group (*n* = 8, *P* < 0.01). However, neither AVE0991 nor A-779 alone had a significant effect on ROS expression.

### 3.2. Effects of AVE0991 on Ang II-Induced VSMC Proliferation and ROS Production with Pretreatment of HO-1 Inhibitor ZnPPIX

**Figure 5:**
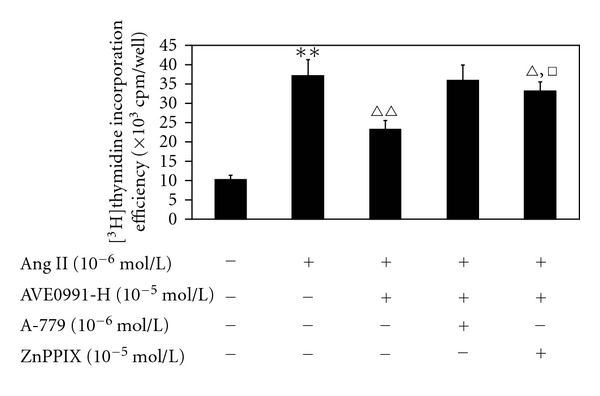
The effect of AVE0991 on the Ang II-induced VSMCs [^3^H]thymidine incorporation efficiency with pretreatment with the HO-1 inhibitor ZnPPIX. **P < 0.01 versus control group; ^*▵*^ *P* < 0.05; ^*▵▵*^ *P* < 0.05 versus Ang II group; ^□^ *P* < 0.01 versus Ang II + AVE0991 group; *n* = 8.

In this experiment, in the Ang II + AVE0991 + ZnPPIX group, the [^3^H]thymidine incorporation efficiency of the VSMCs that were pretreated with ZnPPIX increased significantly when compared to the Ang II + AVE0991 (10^−5^ mol/L) group (*n* = 8, *P* < 0.01), although the efficiency was lower than the Ang II group (*n* = 8, *P* < 0.05) (Figure 5).

**Figure 6:**
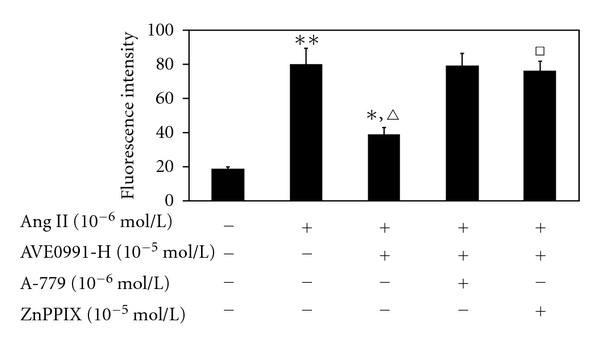
The effect of AVE0991 on Ang II-stimulated ROS production in VSMCs that were pretreated with the HO-1 inhibitor ZnPPIX. *P < 0.05; **P < 0.01 versus control group; ^*▵*^ *P* < 0.01 versus Ang II group; ^□^ *P* < 0.01 versus Ang II + AVE0991 group; *n* = 8.

 To determine whether AVE0991 inhibits Ang II-stimulated ROS production via modulating HO-1 expression, we pretreated VSMCs with the HO-1 inhibitor ZnPPIX (10^−5^ mol/L). The ROS level was significantly higher in the Ang II + AVE0991 (10^−5^ mol/L) + ZnPPIX group compared to the Ang II + AVE0991 (10^−5^ mol/L) group (*n* = 8, *P* < 0.01). However, this AVE0991-mediated effect was also abolished by the Ang-(1–7) Mas receptor antagonist A-779 (Figure 6).

### 3.3. Effect of AVE0991 on p38 Phosphorylation

**Figure 7:**
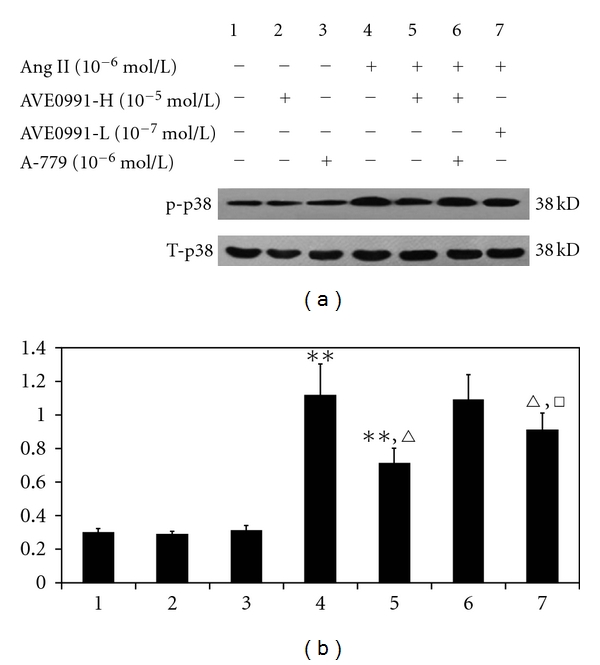
The effect of AVE0991 on the p38 phosphorylation level in VSMCs that were induced by Ang II. Lanes 1–7 represent the control, AVE0991, A-779, Ang II, Ang II + AVE0991-H, Ang II + AVE0991-H + A-779, and the Ang II + AVE0991-L group. **P < 0.01 versus control group; ^*▵*^ *P* < 0.01 versus Ang II group; ^□^ *P* < 0.01 versus Ang II + AVE0991-H group; *n* = 8.

The phosphorylation of p38 was significantly higher in the Ang II (10^−6^ mol/L) group compared to the control group (*n* = 8, *P* < 0.01) (Figure 7). AVE0991 treatment attenuated this increase in p38 phosphorylation, and attenuation of p38 phosphorylation was more pronounced in the AVE0991 higher-concentration treatment group (AVE0991-H, 10^−5^ mol/L) compared to the AVE0991 low-concentration treatment group (AVE0991-L, 10^−7^ mol/L). However, when the cells were treated with Ang II + AVE0991 and 10^−6^ mol/L of A-779, there was no change in the level of p38 phosphorylation compared to the Ang II + AVE0991 group.

### 3.4. Effect of AVE0991 on HO-1 Protein Expression

**Figure 8:**
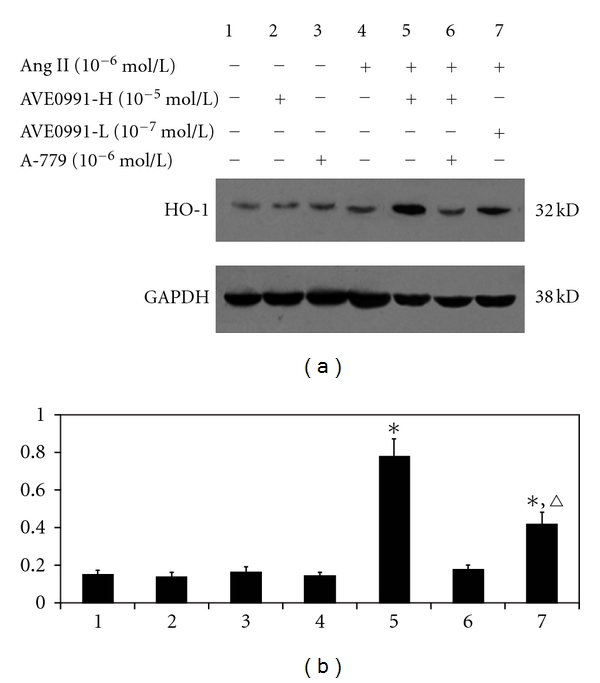
The effect of AVE0991 on HO-1 protein expression in VSMCs that were induced by Ang II. *P < 0.01 versus control group; ^*▵*^ *P* < 0.01 versus Ang II + AVE0991-H group; *n* = 8.

HO-1 protein expression was not significantly different between the Ang II (10^−6^ mol/L) and control groups (Figure 8). However, AVE0991 treatment significantly increased VSMCs HO-1 protein expression in the Ang II + AVE0991 group when compared to the control group (*n* = 8, *P* < 0.01). This increase in HO-1 protein expression was more pronounced in the AVE0991-H treatment group (10^−5^ mol/L) compared to the AVE0991-L treatment group (10^−7^ mol/L) (*n* = 8, *P* < 0.05). However, when the cells were treated with Ang II + AVE0991 (10^−5^ mol/L) + A-779 (10^−6^ mol/L), there was no change in HO-1 expression compared to the Ang II + AVE0991 (10^−5^ mol/L) group. Neither AVE0991 nor A-779 treatment alone has a significant effect on HO-1 protein expression.

### 3.5. Effect of AVE0991 on p38 Protein Phosphorylation When the VSMC Were Pretreated with the HO-1 Inhibitor ZnPPIX

**Figure 9:**
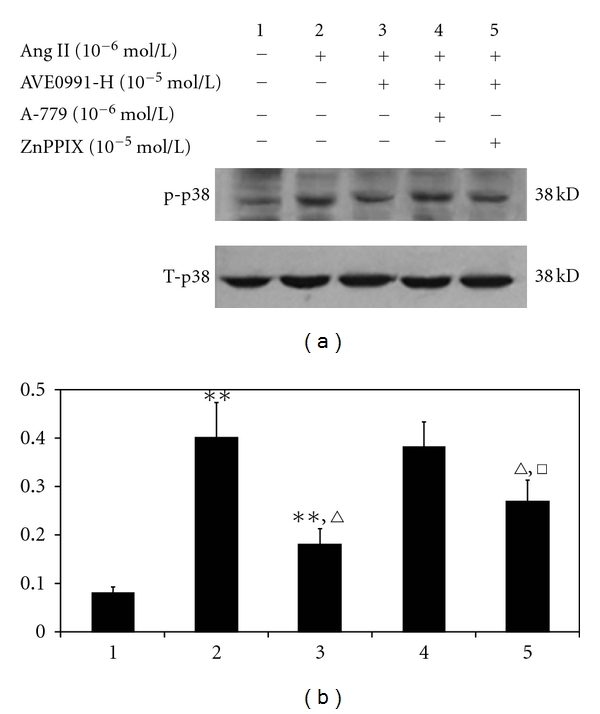
The effect of AVE0991 on the p38 phosphorylation level in VSMCs when combined with the HO-1 inhibitor ZnPPIX. **P < 0.01 versus control group; ^*▵*^ *P* < 0.01 versus Ang II group; ^□^ *P* < 0.01 versus Ang II + AVE0991 group; *n* = 8.

We next sought to determine whether AVE0991 modulates p38 phosphorylation via HO-1 expression. As Figure 9 indicates, the level of p38 phosphorylation in the Ang II (10^−6^ mol/L) group was significantly higher than that in the control group. AVE0991 treatment (10^−5^ mol/L) significantly inhibited Ang II-mediated p38 phosphorylation (*n* = 8, *P* < 0.01), although p38 phosphorylation was higher than that in the control group (*n* = 8, *P* < 0.01). However, when the cells were pretreated with the HO-1 inhibitor ZnPPIX (10^−5^ mol/L), the p38 phosphorylation level in the Ang II + AVE0991 + ZnPPIX group was significantly higher than that in the Ang II + AVE0991 group (*n* = 6, *P* < 0.01), although the phosphorylation level was lower than that in the Ang II group.

## 4. Discussion

The major finding of this study is that AVE0991, a nonpeptide analog of Ang (1–7), attenuates Ang II-induced VSMCs proliferation by inducing heme oxygenase-1 expression and by downregulating p-38 MAPK phosphorylation. In addition, the present study shows that AVE0991 suppresses the Ang II-stimulated ROS production in VSMCs. Furthermore, experiments with the HO-1 inhibitor ZnPPIX indicate that AVE0991 decreases p-38 MAPK phosphorylation via induction of HO-1 protein expression in Ang II-induced VSMCs proliferation. In addition, treatment with AVE0991 attenuated proliferation of the VSMCs in a dose-dependent manner. However, all of the beneficial effects of AVE0991 were completely blocked by pretreatment with the Ang-(1–7) receptor antagonist A-779.

AVE0991 mimics many of the biological actions of Ang-(1–7) and acted like an Ang-(1–7) Mas receptor agonist in Mas-knockout mice and Mas-transfected cells [1, 2, 6, 21]. Ang-(1–7) has been shown to improve vascular endothelial dysfunction, delay the development of cardiac hypertrophy, and attenuate the development of heart failure [3, 22]. EJ Freeman reported that Ang-(1–7) pretreatment also inhibited the proliferative effects of Ang II-treated VSMCs as measured by [^3^H]leucine incorporation and [^3^H]-Thymidine incorporation [19]. Thus, Ang-(1–7) interacts with specific receptors on VSMCs to exert antiproliferative effects that can reverse the Ang II-mediated effects [19]. However, because it is a peptide that is rapidly degraded when orally administered, Ang-(1–7) has limited clinical use. In contrast, AVE0991, the nonpeptide analog of Ang-(1–7), is resistant to proteolytic enzymes and can thus be clinically oral applied to treat cardiovascular and related diseases.

Previous studies have shown that Ang II plays an important role in VSMCs proliferation [10, 12]. In vitro, Ang II is one of the most important factors that contribute to VSMCs proliferation by increasing protein and DNA synthesis through the type 1 Ang II receptor [23]. Because Ang II induces a significant increase in VSMCs protein synthesis in conditioned medium [24], the VSMCs were starved in DMEM medium without FBS for 36 h in our experiments to minimize the medium-induced myocyte proliferative effects. According to our previous studies and other published results, the cells were starved for 48 hours prior [^3^H]-Thymidine incorporation experiments [19]. In addition, a VSMCs purity of greater than 98% was controlled in every experiment group to decrease false results that could be caused by contamination.

The present study indicates that Ang II promotes VSMCs DNA synthesis (measured via [^3^H]-Thymidine incorporation) in vitro compared to the control group, which agrees with the previously published findings. Treatment with AVE0991 + Ang II significantly suppressed DNA synthesis. Moreover, when the concentration of AVE0991 was increased from 10^−8^ mol/L to 10^−5^ mol/L, AVE0991 inhibited the Ang II-mediated increase in DNA synthesis of the VSMCs in a dose-dependent manner. When the cells were pretreated with the Ang-(1–7) Mas receptor antagonist A-779, the beneficial effect of AVE0991 was completely abolished, which also suggested that the AVE0991 is a nonpeptide Mas receptor agonist. In addition, treatment with AVE0991 alone significantly suppressed VSMCs DNA synthesis. Conversely, A-779 alone also elicited a significant increase in DNA synthesis. However, differently to the results that were seen with DNA synthesis, neither treatment with AVE0991 alone nor treatment with A779 alone can alter ROS production and p38 phosphorylation. Future studies are needed to confirm these results.

Although AVE0991 treatment is antiproliferative in VSMCs, the mechanisms of this effect remain unclear. Previous studies have shown that Ang II activates the NAD(P)H oxidase enzyme system and promotes the generation of ROS, such as the superoxide anion and hydrogen peroxide which stimulate smooth muscle cell proliferation [10, 11, 25]. In addition, the mitogen-activated protein kinase cascade, particularly p38 MAPK, may be an important intracellular mediator of responses that are related to cell growth and differentiation [18]; Ang II is a well-known activator of this signaling pathway. According to this evidence, we question whether AVE0991 inhibits Ang II-stimulated VSMCs proliferation by modulating ROS production or by modulating the p38 pathway. In our study, Ang II promoted ROS production compared to the control group. However, treatment with AVE0991 significantly suppressed Ang II-mediated ROS production in a dose-dependent manner. Similarly, the nonpeptide AVE0991 also inhibited Ang II-mediated phosphorylation of p38MAPK. Nevertheless, pretreatment with the Ang-(1–7) Mas receptor antagonist A-779 also abolished the inhibitory effects of AVE0991. Hence, the present study shows that AVE0991 may inhibit Ang II-mediated VSMCs proliferation by decreasing ROS production and altering the p38 pathway. However, as an important intracellular second messenger, ROS can activate many downstream signaling molecules, including MAPK; thus, there may be a relationship between the ROS and p38 mechanisms of AVE0991. Nevertheless, more direct evidence should be established to fully understand these mechanisms.

Recently, induction of heme oxygenase- (HO-) 1 expression in vivo has been reported to suppress NADPH oxidase-derived oxidative stress. Additionally, HO-1 overexpression suppressed the Ang II-induced hypertrophic response in cardiomyocytes via decreasing the ROS production stress [20]. Ang II-stimulated growth of VSMCs has an essential redox-sensitive component that is mediated by activation of the MAPK-dependent signaling pathways [26]. In addition, HO-1 attenuates Ang II-induced VSMCs proliferation that involves MAPK inhibition [18]. In neutrophils, HO-1 attenuates infiltration during sepsis via inactivation of p38 MAPK [27]. Hence, we wonder whether there is any relationship between HO-1 and p38 MAPK in Ang II-induced VSMCs proliferation with AVE0991 treatment. In the present study, the nonpeptide AVE0991 dose-dependently increased HO-1 protein expression in the Ang II + AVE0991 group. However, Ang II alone has no significant effect on HO-1 protein expression, which agrees with the previous data in cardiomyocytes [20]. In addition, pretreatment with the HO-1 inhibitor ZnPPIX significantly attenuated the inhibitory action of AVE0991 on Ang II-induced VSMCs DNA synthesis, which indicates that AVE0991 inhibits Ang II-induced VSMCs proliferation partly via induction of HO-1. On the other hand, ZnPPIX pretreatment significantly increased the Ang II-induced p38 phosphorylation level. This indicates that AVE0991 attenuates phosphorylation of p38 partly via induction of HO-1 expression in Ang II-induced VSMCs proliferation.

In summary, our results suggest that the ACE2-Ang-(1–7)-Mas pathway may play a more important antiproliferative role than our current understanding of endogenous Ang-(1–7) in RAS. Moreover, Ang II treatment upregulates p38 phosphorylation and ROS production, which contribute to VSMCs proliferation. Treatment with AVE0991 attenuates Ang II-induced VSMCs proliferation in a dose-dependent manner, which may be associated with regulation of Mas/HO-1/p38 signaling pathway.
